# Human protein-coding genes and gene feature statistics in 2019

**DOI:** 10.1186/s13104-019-4343-8

**Published:** 2019-06-04

**Authors:** Allison Piovesan, Francesca Antonaros, Lorenza Vitale, Pierluigi Strippoli, Maria Chiara Pelleri, Maria Caracausi

**Affiliations:** Unit of Histology, Embryology and Applied Biology, Department of Experimental, Diagnostic and Specialty Medicine (DIMES), University of Bologna, Bologna, BO Italy

**Keywords:** Human genes, Protein-coding genes, Gene statistics

## Abstract

**Objective:**

A well-known limit of genome browsers is that the large amount of genome and gene data is not organized in the form of a searchable database, hampering full management of numerical data and free calculations. Due to the continuous increase of data deposited in genomic repositories, their content revision and analysis is recommended. Using GeneBase, a software with a graphical interface able to import and elaborate National Center for Biotechnology Information (NCBI) Gene database entries, we provide tabulated spreadsheets updated to 2019 about human nuclear protein-coding gene data set ready to be used for any type of analysis about genes, transcripts and gene organization.

**Results:**

Comparison with previous reports reveals substantial change in the number of known nuclear protein-coding genes (now 19,116), the protein-coding non-redundant transcriptome space [now 59,281,518 base pair (bp), 10.1% increase], the number of exons (now 562,164, 36.2% increase) due to a relevant increase of the RNA isoforms recorded. Other parameters such as gene, exon or intron mean and extreme length appear to have reached a stability that is unlikely to be substantially modified by human genome data updates, at least regarding protein-coding genes. Finally, we confirm that there are no human introns shorter than 30 bp.

## Introduction

A well-known limit of genome browsers [1–3] is that the large amount of data they provide about human genome and genes is not organized in the form of a searchable database [4], hampering a full management of numerical data and free calculations on data subsets. We have previously shown that GeneBase, a software with a graphical interface able to import and elaborate data available in the National Center for Biotechnology Information (NCBI) Gene database, allows users to perform original searches, calculations and analyses of the main gene-associated meta-information [5], and since the release of GeneBase 1.1, it can also provide descriptive statistical summarization such as median, mean, standard deviation and total for many quantitative parameters associated with genes, gene transcripts and gene features for any desired database subset [6].

Due to the continuous increase of data deposited in genomic repositories, a revision and analysis of their content is recommended. We provide here a tabulated set of data about human nuclear protein-coding genes that may be useful for human genome studies and analysis. While the basic approach to obtain the data we present here is similar to the one followed in our previous study about the subject [6], there are two main differences. First, the data are now updated as of January 2019 rather than January 2016, exploiting novel information made available in the last 3 years and thus showing how some parameters have been subjected to relevant changes, while others appear to be stable.

In addition, following analysis based on the relationships between different data tables provided by the database at the core of the GeneBase tool, we provide the results in the simple form of a spreadsheet table, providing three data sets ready to be used for any type of analysis of the data about nuclear protein-coding genes, transcripts and gene organization (exons, coding exons and introns). In order to provide reliable data, we focused on a curated subset of human nuclear protein-coding genes with a REVIEWED or VALIDATED Reference Sequence (RefSeq) status [1, 7]. The reasons for the choice of the NCBI Gene database as a reference data source have been previously discussed in detail [6].

Main summarized data derived from the analysis of our updated and standard-formatted data sets are also provided here, while the data tables remain available for human genome studies.

## Main text

### Database search and data import

All the currently (alive/live qualification) available human nuclear gene entries were downloaded from NCBI Gene web site on January 5th, 2019 using the following text query: “Homo sapiens” [Organism] AND “source_genomic” [properties] AND alive [property].

The resulting file has been imported according to the user guide of GeneBase 1.1, available for free at http://apollo11.isto.unibo.it/software/ and including a FileMaker Pro runtime (FileMaker, Santa Clara, CA) at its core. Python scripts provided with the software were run for the initial data pre-processing. The downloading, parsing and import of gene entries are described in more detail in the software public documentation.

### Database searching and export

In order to provide a curated set of updated statistics regarding human nuclear protein-coding genes and transcripts through GeneBase 1.1 Human, we considered only NCBI Gene records retrieved by searching for protein-coding gene type, with REVIEWED or VALIDATED RefSeq gene status, with at least one REVIEWED or VALIDATED transcript, excluding records annotated as “not in current annotation release” records (Genome_Annotation_Status field).

This selection retrieved 19,116 genes, 46,932 transcripts and 562,164 exons. The data sets were created by exporting the data from each relative table of GeneBase as a spreadsheet. Thus, three tables in the open standard format .xlsx (Microsoft, Seattle, WA), Genes.xlsx, Transcripts.xlsx and Gene_Table.xlsx, are provided here. The description of each field is included in the first row of the spreadsheet table.

Data in the Genes.xlsx table are NCBI Gene identifier, official Gene Symbol, Chromosome, Gene Type, gene RefSeq status, transcript RefSeq status, Gene Length in bp. They were derived from the GeneBase “Genes” table, including official Gene Symbol, Chromosome, Gene Type, and gene RefSeq status from the “Gene_Summary” related table. Chromosome values were re-exported from GeneBase in text format and pasted into the relative column of Genes.xlsx file to avoid misinterpretation of “X” and “Y” values as numbers by Excel.

Data in the Transcripts.xlsx table include the same first five types of information provided in the Genes.xlsx table, plus RefSeq GenBank accession number for each transcript, length in bp of the whole transcript as well as of its 5′ untranslated region UTR, coding sequence (CDS) and 3′ UTR, number of exons and coding exons for that transcript, derived from the GeneBase “Transcripts” table.

Data in the Gene_Table.xlsx table are derived from the “Gene Table” section of the NCBI Gene resource parsed by GeneBase “Gene_Table” table and include, along with NCBI Gene identifier, official Gene Symbol and Gene Type, along with data about each gene exon/intron represented in each row: chromosome sequence RefSeq GenBank accession number, start and end coordinates, chromosome strand and length in bp for the gene to which the exon/intron belongs; length in bp for the relative transcript; coordinates and length in bp of the 5′ UTR, CDS and 3′ UTR of the transcript to which the exon/intron belong; RefSeq status, label and GenBank accession number for that transcript; start and end coordinates, length in bp and serial number for each exon, coding exon and intron; last exon annotation which shows “Yes” if that exon or coding exon is the last in the transcript; protein RefSeq label and GenBank accession number; non-redundant annotation, which shows “Yes” to label each exon/coding exon/intron a single time (“Yes—Merged” meaning that the same element appears to be repeated in the data, “Yes—Unique” meaning that the element is unique in the data set); live status, genome annotation status and gene RefSeq status for the gene derived from the GeneBase “Gene_Summary” related table. Filtering by the “Yes” annotation allows the retrieval of a non-redundant set of exons, coding exons and introns, respectively. Intron data are presented as companions to the relative upstream exon, there will therefore be no intron data in the rows with Last_Exon field showing “Yes”.

We have generated general descriptive statistics for human nuclear protein-coding genes and messenger RNAs (mRNAs) (Table 1), exons, coding-exons and introns (Table 2).

**Table 1:** Number and length of known human nuclear protein-coding genes and protein-coding transcripts (mRNAs)

	Protein-coding genes^a^	mRNAs^b^
Number		
Total entries	19,116	49,632
Median	N/A	N/A
Mean	Per chr: 797	N/A
SD	N/A	N/A
Min	chrY: 47 chr21: 228	N/A
Max	chr1: 1952	N/A
Length		
Median	26,018 bp	2938 bp
Mean	66,646 bp	3522 bp
SD	131,781 bp	2557 bp
Shortest	189 bp (*KRTAP6*-*2*, chr21)	186 bp (*DEFB133*, chr6)
Longest	2,473,592 bp (*RBFOX1*, chr16)	109,224 bp (*TTN*, chr2)
Total	1,274,002,474 bp	174,797,813 bp

*SD* standard deviation, *chr* chromosome, *min* minimum, *max* maximum, *bp* base pair

^a^Values of protein-coding genes have been calculated exploiting Excel functions in Genes.xlsx file containing data exported from GeneBase “Genes” and “Gene_Summary” tables (records retrieved searching for nuclear protein-coding gene type and REVIEWED or VALIDATED gene RefSeq status and REVIEWED or VALIDATED transcript RefSeq status, excluding records annotated as “not in current annotation release”). Min and max number of genes per chr were derived using filter function in the Excel Genes.xlsx file. Mean number per chr has been calculated dividing the total number of genes by 24 (22 autosomes, chrX and chrY)

^b^Values were calculated exploiting Excel functions in Transcripts.xlsx file containing data exported from GeneBase “Transcripts” table (retrieved records with a VALIDATED or REVIEWED RefSeq status with an “NM_” type of corresponding RefSeq RNA accession number belonging to genes with a VALIDATED or REVIEWED RefSeq status, excluding “not in current annotation release” records). The gene locations have been retrieved manually from GeneBase “Gene_Summary” table. N/A: not applicable

**Table 2:** Number and length of human exons and introns in protein-coding transcripts

	Exons (E)	Coding exons^a^	Introns (I)
Number			
Total entries	562,164	512,303	512,530
Total non-redundant entries	159,652	151,285	148,092
Median per transcript	9.0	8.0	8.0
Mean per transcript	11.3	10.3	10.3
SD per transcript	9.6	9.6	8.6
Min per transcript	1 (1074 transcripts; 1068 genes)	1 (3157 transcripts; 2117 genes)	1 (1960 transcripts; 1572 genes)
Max per transcript	363 (*TTN*, chr2)	362 (*TTN*, chr2)	362 (*TTN*, chr2)
Length			
Median	131 bp Not last^b^: 124 bp	120 bp	1747 bp
Median non-redundant	142 bp Not last^b^: 130 bp	121 bp	1742 bp
Mean	311 bp Not last^b^: 159 bp	160 bp	6938 bp
Mean non-redundant	371 bp Not last^b^: 177 bp	171 bp	7397 bp
SD	744 bp Not last^b^: 205 bp	254 bp	22,163 bp
SD non-redundant	828 bp Not last^b^: 242 bp	293 bp	24,263 bp
Shortest	2 bp (*GRK6*, E16; *SEPT7*, E2)	1 bp (e.g., *GSTP1*, last base of E1)	26 bp (*XBP1*, I4) 30 bp (*RBP5*, I2 and *MST1L,* I9)
Longest	27,303 bp (*GRIN2B*, E13, last, with 1857 coding bp)	21,693 bp (*MUC16*, E3)	1,160,411 bp (*ROBO2*, I2)
Total	174,797,813 bp	82,144,360 bp	3,555,747,074 bp
Total non-redundant	59,281,518 bp	25,840,698 bp	1,095,434,245 bp

Median, mean, SD, min and max number of exons or coding exons per transcript were calculated exploiting Excel functions in Transcripts.xlsx file (containing data exported from GeneBase “Transcripts” table, i.e. retrieved records with a VALIDATED or REVIEWED RefSeq status with an “NM_” type of corresponding RefSeq RNA accession number belonging to genes with a VALIDATED or REVIEWED RefSeq status, excluding “not in current annotation release” records). Number of introns per transcript was estimated assuming: (number of exons—1). Minimum number of introns per transcript was found excluding mono-exonic genes. Number of genes with one exon can be retrieved filtering Excel rows for Exons_per_RNA equal to 1, copying the retrieved gene symbols in a new sheet and applying the Excel “Advanced Filter” called “Unique records only”. Number of genes with one intron can be found with the same procedure, filtering Excel rows for Exons_per_RNA greater than 1. Length values were calculated exploiting Excel functions in Gene_Table.xlsx file containing data exported from GeneBase “Gene_Table” table (retrieved as above). When calculations were performed on filtered data, “AGGREGATE” Excel function was used. Exon and intron non-redundant sets were found counting only one exon or intron for each group of exons or introns present in multiple transcript isoforms, i.e. filtering for Excel rows containing “Yes” in the relative Non_Redundant column. Values were calculated for the total number of entries when “non-redundant” is not specified. Total number of entries was calculated in Gene_Table.xlsx file using Excel “Count number” function for each column containing length_bp values, filtering to select non-redundant entries when indicated. Total length for each feature was calculated in Gene_Table.xlsx file using Excel “Sum” function for each column, filtering to select non-redundant entries when indicated

*SD* standard deviation, *min* minimum, *max* maximum, *chr* chromosome, *bp* base pair

^a^In this column numbers and lengths are shown considering only the protein-coding portion of exons, including stop codons

^b^These values were calculated excluding records corresponding to the last exon, which is usually the longest one, filtering for Excel rows not containing “Yes” in Last_Exon column

### Data validation

The data presented in the Genes.xlsx, Transcripts.xlsx and Gene_Table.xlsx have been counter-checked with the complete, original data included in the GeneBase software. Using the spreadsheet filtering and summarization functions (Excel for Mac 2011, Microsoft) or exploiting the search and calculation functions in GeneBase (FileMaker Pro) provided identical results in all cases.

Following validation by the software Splign [8], we confirm that there are no human (and possibly of any species) introns shorter than 30 bp (Table 2). Actually, apart from three introns estimated to be of 1–3 bp long due to NCBI Gene “Gene Table” artifacts [5], there is one unique intron smaller than 30 bp, intron 14 of *XBP1* gene, in these data. However, rather than an intron excised via canonical splicing, this is a 26-nucleotide segment known to be removed in particular circumstances by a completely different mechanism, an excision mediated by the endonuclease inositol-requiring enzyme 1 (IRE1) [9].

## Discussion

Here we provide a tabulated set of data about human nuclear protein-coding genes (genes, transcripts and gene features such as exons, coding portion of the exons and introns) derived from advanced parsing of NCBI Gene web site offered in a standard, ready-to-use spreadsheet format. The data are updated as of January 2019, 3 years after the last published analysis of human gene features [6] and pre-filtered according to public annotation about the review or validation of the records to ensure reliability of the data.

The availability of the data sets presented here allows a ready update of main parameters about human genome, often cited in textbooks or reports without a source accounting for a rigorous method for extracting this information.

Comparison with a previous report of 3 years ago [6], which in turn demonstrated important differences with the first analysis of the human genome sequence [10, 11], reveals some substantial changes in relevant parameters such as the number of known, characterized nuclear protein-coding genes (from 18,255 to 19,116), thus now approaching a limit theorized 5 years ago [12]; the protein-coding non-redundant transcriptome space (from 53,827,863 to 59,281,518 bp, with an increase of 10.1%); number of exons (from 412,641 to 562,164, plus 36.2%, when this number is not collapsed to eliminate redundant exons appearing in more than one mRNA) due to a relevant increase of the number of mRNA isoforms recorded.

Regarding the number of genes, it should in any case always be kept in mind that positive, but not negative, evidence for the existence of a gene may be obtained because, from a structural point of view, a locus could be present, or amplified, due to a copy number variation (CNV) shared by only a limited number of subjects. On the other hand, a genetic element could be transcribed, and thus identified as a functional gene, only under particular conditions such as a developmental stage, a disease or the exposure to specific stresses or drugs. Therefore, in the end the actual overall number of functional genes will always be subject to a continuous update and refinement.

Other parameters such as exon/intron mean and extreme length appear to have reached a stability that is unlikely to be substantially modified by future updates of the human genome data, which appear to be approaching a plateau on the curve of new added data, at least where protein-coding genes are concerned [6].

The data sets are provided in standard, open format.xlsx. Following the opening of the data sets in a spreadsheet application, users have easy access to the whole set of current reviewed/validated data about human nuclear protein-coding genes. It is possible to use calculation and statistical functions of the spreadsheet to analyze the data in any direction. The spreadsheets we provide allow the immediate identification of key features of genes or gene elements by simply filtering or ordering the data sets, the access to mRNA data already split to highlight 5′ UTR, CDS and 3′ UTR and an easy export or import of the data for any further analysis, as for instance general descriptive statistics for human nuclear protein-coding genes and mRNAs, exons, coding-exons and introns summarized here.

In addition, data can be exported in other formats and imported in other applications (database management systems, statistical software, genomic tools) for further analysis. For instance, it would easily become possible to explore hypotheses about the correlation of structural details of human nuclear protein-coding genes to their level of expression, exploiting quantitative descriptions of the human transcriptome [13], or to the dosage of metabolites related to enzyme proteins, exploiting quantitative representations of human metabolome in health and disease [14].

In addition, statistics based on these data and any subset generated from them may be used to tune genomic software requiring parameters about nuclear protein-coding gene, transcript or exon/intron number and length [15, 16].

These data might also be used in comparative genomic studies when compared to similar data sets generated from different species to uncover specific and significant differences in genome and gene organization.

Finally, these data might be useful to design experiments for poorly characterized human genome regions, as in, for example, our current annotation effort of the recently defined highly restricted Down Syndrome critical region (HR-DSCR), which to date does not contain known genes [17], or to study transcription mechanisms such as alternative splicing or nonsense-mediated messenger RNA decay.

## Limitations

All these kinds of analyses depend on the chosen gene entry subset, the RefSeq classification system and are subject to the accuracy of the input dataset.

## Data Availability

The three data tables Genes.xlsx, Transcripts.xlsx and Gene_Table.xlsx have been released in the public repository Open Science Framework and they can be freely downloaded at the address: https://osf.io/mhda7/.

## Abbreviations

NCBI: National Center for Biotechnology Information
RefSeq: Reference Sequence
UTR: untranslated region
CDS: coding sequence
mRNA: messenger RNA
IRE1: inositol-requiring enzyme 1
CNV: copy number variation
HR-DSCR: highly restricted Down Syndrome critical region
